# Morphological and physiological response of sour orange (*Citrus aurantium* L.) seedlings to the inoculation of taxonomically characterized bacterial endophytes

**DOI:** 10.1016/j.sjbs.2022.01.051

**Published:** 2022-01-29

**Authors:** Sehrish Mushtaq, Muhammad Shafiq, Muhammad Saleem Haider, Gulzar Ahmad Nayik, Saleh H. Salmen, Hesham Ali El Enshasy, Ahmed Atta Kenawy, Gulden Goksen, Edgar Vázquez-Núñez, Mohammad Javed Ansari

**Affiliations:** a Faculty of Agricultural Sciences (IAGS), Department of Plant Pathology, University of the Punjab, Lahore,-54000 Pakistan.; b Faculty of Agricultural Sciences (IAGS), Department of Horticulture, University of the Punjab, Lahore-54000 Pakistan.; c Department of Food Science & Technology, Govt. Degree College, Shopian 192303, J&K, India; d Department of Botany and Microbiology, College of Science, King Saud University, PO Box 2455, Riyadh 11451, Saudi Arabia; e Institute of Bioproduct Development (IBD), Universiti Teknologi Malaysia, 81310 Johor Bahru, Malaysia; f School of Chemical and Energy Engineering, Faculty of Engineering, Universiti Teknologi Malaysia, 81310 Johor Bahru, Malaysia; g City of Scientific Research and Technology Applications (SRTA), New Burg Al Arab, Alexandria, Egypt; h Food Technology Department, Vocational School of Technical Sciences at Mersin Tarsus Organized Industrial Zone, Tarsus University, 33100, Mersin, Turkey; i Department of Chemical, Electronic and Biomedical Engineering, Division of Sciences and Engineering, University of Guanajuato, Lomas del Bosque 103, Lomas del Campestre, Leon, Guanajuato C.P. 37150, Mexico; j Department of Botany, Hindu College Moradabad (Mahatma Jyotiba Phule Rohilkhand University), Bareilly, UP 244001, India

**Keywords:** Plant-microbe interaction, Physiology, Endophytes, Inoculation methods, Sour orange, 16S rRNA

## Abstract

Entophytic bacteria (EBs) are very diverse and found in virtually all plant species studied. These natural EBs live insides the host plant and can be used to maximize crop and fruit yield by exploiting their potential. In this paper, EBs characterization from various citrus genotypes and their influence on the morphological and physiological functioning of sour orange (*Citrus aurantium*) seedlings are described. To assess the influence of 10 distinct EBs, three different techniques (injection, soil mix, and spray) were applied for single and mixed inoculation on sour orange (*C. aurantium*) seedlings. The selected strains were identified as firmicutes (*Enterococcus faecalis, Bacillus safensis, Bacillus cereus, Bacillus megaterium, Brevibacillus borstelensis* & *Staphylococcus haemolyticus),* and gamma Proteobacteria (*Enterobacter hormachaei, Proteus mirabilis, Pseudomonas aeruginosa,* & *Pseudomonas* sp.) by 16S rRNA gene sequencing. To investigate the influence of these EBs on host plant morphology, different parameters (morphometric) were recorded after five WOI (weeks of inoculation), including shoot/root length, shoot/root fresh and dry biomass, and biophysical analyses i.e., relative water content (RLWC). Physiological markers such as chlorophyll & carotenoid content, protein content, proline content, phenolics, and flavonoids were also analyzed to determine the influence of endophytes on sour orange seedlings. Five strains such as SM-34, SM-20, SM-36, SM-68, and SM-56 significantly improved the development and physiology of sour orange seedlings. *Bacillus cereus* and *Pseudomonas aeruginosa* produced the best outcomes in terms of plant growth. The relative quantification of bacterial inoculums was determined using real-time PCR. A rise in the number of bacterial cells in inoculated treatment suggests that bacterial strains survived and colonized successfully, and also shown their competitiveness with native bacterial community structure. As per the results of inoculation methods, soil mixing, and injection methods were determined to be effective for bacterial inoculation to plants but a variable trend was found for different parameters with test bacterial strains. After testing their impact on field conditions, these strains can be applied as fertilizers as an alternative to conventional chemical fertilizer, although in the context of mixed inoculation of bacterial strains, 5 M and 6 M performed best and enhanced plant growth-promoting activity.

## 1 Introduction

Plant-microorganism interactions are highly complicated, and mainly dependent on microbes and environmental circumstances that affect the plant's physiological status (Moutia et al., 2010). Microbes are ubiquitous in open fields, which can minimize/mask the impact of inoculants (Rosenblueth and Martínez-Romero, 2006). Plant growth regulating (PGRB) bacteria can colonize shoots, roots, leaves, and flowers without apparent symptoms (Compant et al., 2005, Ali et al., 2017). Plant EBs interactions have been assessed for increasing plant yield and dry matter improvement (Botta et al., 2013). This improvement can be linked with improve nutrition in EBS inoculated plants but the response of different EBs in different plant species are different in the presence of the natural environment (De Oliveira et al., 2006). Similarly, the single or mixed EBs inoculum has a different impact on plant physiology. For example, inoculating diverse strains of EBs in the mixture increased more tomato yield in contrast to a single infection, which was explained by increased intake of nitrogen and phosphorus (Botta et al., 2013).

These interactions may improve plant nutrient utilization by enhancing root development, nitrate uptake, or phosphorus solubilization, as well as controlling soil-borne diseases (Patel and Minocheherhomji, 2018). EBs have a variety of beneficial impacts on host plants, such as enhancing plant growth, N2 fixation, and inducing plant disease resistance, among these factors (Patle et al., 2018, Rosenblueth and Martínez-Romero, 2006). EBs produce a wide range of bio-stimulant, and other secondary metabolites with distinct structures (Gaiero et al., 2013). EBs often provide a substance to the plant that is generated by the bacteria in exchange for facilitating the uptake of nutrients from the microenvironment. Increases in germination rates, root growth, yield (Shameer and Prasad, 2018), magnesium, nitrogen, leaf area, shoot/root weights, chlorophyll as well as protein content, hydraulic activity, drought (salt stress tolerance), delayed leaf senescence are just a few of the plant growth benefits caused by the addition of PGPR (Lucy et al., 2004, Calvo et al., 2014). The microbiome of plants is made up of several microbial communities that live in the roots, shoots, and endosphere. Endophytic microorganisms have recently received more attention due to their close relationship with the host (Hardoim et al., 2015); it is thought that plant phytochemical constituents are linked to endophytic bacteria and their interaction with hosts, either direct or indirect (Varma et al., 2017). Inoculation of shrubs and trees, vegetables, or crops with PGPR has been shown to improve shoot weight, plant height, plant vigor, seedlings germination, nutritional and improved nodule formation in legumes (Saharan and Nehra, 2011).

Understanding how plants react to bacterial inoculation and what processes are activated is critical for optimizing the use of bacteria as an alternative technology to boost plant growth and output (Landell et al., 2005). It is now obvious whether endophytic bacteria combinations would benefit citrus, to answer the following question: do endophytic bacteria increase the physiology and growth of citrus in a genotype-dependent manner? In terms of community, it is unclear if bacterial endophytes benefit from remaining within plant tissues rather than growing freely in the space surrounding plant roots (Rosenblueth and Martínez-Romero, 2006). What appears to be evident is that endophytes could provide a few benefits to the host plant, such as improving plant growth and pathogen defense; communication, and interaction with the plant more efficiently than rhizobial microbes under a range of stress factors and conditions (Coutinho et al., 2015).

The objectives of this study were to evaluate the effects of inoculation often potent and well-characterized bacterial strains on agronomic and physiological attributes of citrus, under controlled conditions in Lahore, Pakistan. Citrus is enriched with vitamins A & B, ascorbic acid, and minerals such as phosphorus, iron, and calcium, all of which contribute to the nutritional value of the fruit (Boudries et al., 2015). We hypothesized that these test strains are competent enough to exert their growth-promoting effect when exposed to compete with the native microbial diversity of plants.

## 2 Materials and methods

### 2.1 Sampling

Samples of diverse local varieties of *viz*. Musambi, Kinnow, Grapefruit, Lemon, Sweet orange, Dancy citrus reticulate, Olinda Valencia, Sour orange showing symptoms of citrus greening were collected from the citrus orchards of Punjab located at different locations i.e. Sargodha (32.11722°N/72.67667°E), Multan (29.99083°N/72.0325°E), Sahiwal (30.05°N/72.35°E), Mian Chanu (30.43167°N/72.34722°E), Lahore (31.49472°N/ 74.29 611°E) in September 2015 at fruit harvesting stage and preserved in −80 °C. The samples were collected in sterilized polythene bags and transported carefully to the Lab for more examination.

### 2.2 Isolation and identification of bacteria

Isolation of bacteria was carried out by mince soaked method (Sechler et al., 2009). A 4 cm portion of the leaf midrib from every sample was submerged in NaoCl {1% (w/v)} for 3–4 min and washed 3–4 times with sterilizing (ddH2O). The sterilized tissue was mashed and immersed for 10–20 min in distilled water (100–200 mL). Then the suspension was streaked on an LB agar plate with a sterile loop and incubated at 28 °C (Padder, 2016). The next day the isolated colonies were streaked on the new plate of LB agar for purification and incubated for 48 h at 28 ± 2 °C. The complete purification was achieved by repeated streaking and incubation.

### 2.3 Characterization of endophytes by 16SrRNA

DNA was extracted by the CTAB method (Wilson, 1987). Total genomic DNA of ten bacterial strains was subjected to PCR using 16S rDNA primers (universal) 27F (5′ AGAGTTTGATCMTGGCTCAG 3′), 1492R (5′ ACCTTGTTACGACTT 3′) (Roy et al., 2018), and previously reported PCR conditions were applied. 55 °C was used as annealing temperature for 16S rRNA universal Primers amplification and 1500 bp PCR Product was obtained as described by (Mushtaq et al., 2021). All of the PCR products were gel purified and forwarded to Macrogen in South Korea for sequencing. The obtained sequences were aligned with sequences from Gene Bank using BLASTn, and the Ribosomal Database Project (RDP Hierarchy Browser). Sequences were deposited to Gene Bank (Table 1).

**Table 1:** Taxonomic classification of selected bacterial isolates.

Treatments	Codes of Bacterial Strain used in this study	Identified strains (Closest match in Database)	% 16S rRNA identity	Accession numbers
Treatment (1)	SM (1)	*Staphylococcus haemolyticus*	97%	MF957708
Treatment (2)	SM (20)	*Proteus mirabilis*	99%	MF958504
Treatment (3)	SM (27)	*Enterobacter* hormaechei	97%	LT745966
Treatment (4)	SM (34)	*Bacillus safensis*	99%	MF801628
Treatment (5)	SM (36)	*Bacillus cereus*	97%	MF801630
Treatment (6)	SM (42)	*Brevibacillus borstelensis*	93%	LT745989
Treatment (7)	SM (56)	*Bacillus megaterium*	94%	MF802485
Treatment (8)	SM (57)	*Pseudomonas* sp.	97%	MF973203
Treatment (9)	SM (68)	*Pseudomonas aeruginosa*	95%	MF802727
Treatment (10)	SM (76)	*Enterococcus faecalis*	100%	LT844634

### 2.4 Evolutionary tree

Multiple sequence alignments (MSA) and phylogenetic tree (Neighbor-joining Algorithm and 1000 bootstrap) of 16S rDNA gene sequences were performed using MEGA X.0 software (Tamura et al., 2013). A phylogenetic tree was created using to evaluate the evolutionary relationship between organisms.

### 2.5 An experiment in green house

Sour orange seedlings were used to test pathogenicity and host-pathogen interaction of isolated bacterial culture under controlled conditions in a glasshouse. The *C. aurantium* seedlings were sown in (100 × 9 cm) containers filled with a soil mixture (6 kg Clay and Compost). One-year-old plants of Sour orange seedlings were used for EBs (taxonomically characterized bacterial endophytes) inoculum and a biochemical test.

### 2.6 Inoculum preparation and inoculation

EBs Cultures were grown to a concentration of 1 × 10^9^ CFU mL^−1^, centrifuged at 8000g, and rinsed thoroughly with saline (0.85 percent, w/v) before inoculum preparation. The cells were adjusted to 10^8^ CFU mL by resuspending them in equal volumes of saline. Three alternative methods were used to inject one mL of EBs suspension (10^8^ CFU mL^−1^) into *C. aurantium* seedlings: (1) Using a hypodermic needle, inject cell suspension into the leaf's intercellular spaces, (2) spraying inoculum into plant leaf with a spray bottle, (3) incorporating cell suspension into the soil and allowing it to reach the roots. There was one positive control that did not get any inoculum. The experiment was carried out in a greenhouse with three replications of each treatment using a completely randomized design (CRD) (day/night temperature 25 °C, light/dark durations 16/8). After five weeks of inoculation, data on morphological and physiological growth parameters were recorded. The treatments are described in detail in (Table 1).

### 2.7 Measurement of morphological parameters

Morphometric parameters such as SL/RL (cm), SFW/RFW, and dry biomass (g) of sour orange seedlings were measured after one month. Upon plucking sour orange seedlings from the soil, they were dissected into shoots and roots using a sharp blade. From the base to the tip, the length of the shoot was measured in centimeters. Data were obtained for each shoot. The root length was measured from the top to the lowest dripping tip in cm. Individual root measurements were taken and reproduced three times. A digital electric balance was used to measure the fresh weight (g) of 3 replications per treatment. Following the measurement of fresh weights of shoots/roots for each treatment, plant fresh tissues (shoot and root) were dried at 80 °C for 24 h in an oven to obtain dry weight (g) data.

Three types of weights were measured to calculate the relative leaf water content. (1) Fresh weight of leaf, (2) dry weight of leaf, (3) turgid weight of leaf (g). Leaves were removed and weighed on a weighing scale. The leaves were then immersed in water for 8–10 h to determine their turgid weight, after which they were placed in an oven to dry for 24 h at 800 °C. The RWC was calculated using the following formula:

$$\text{The RWC}\;\left(\%\right)\;=\;\left[\left(\text{W}-\text{DW}\right)\;/\;\left(\text{TW}-\text{DW}\right)\right]\;\text{x 1}00$$

where W = Sample fresh weight, TW = Sample turgid weight, and DW = Sample dry weight.

### 2.8 Study of physiological traits

1 g of fresh plant leaves were homogenized in an 80 percent acetone solution with a pestle in a mortar for chlorophyll content determination. The absorbance of chlorophyll *a* and b, as well as carotenoids at wavelengths of (645, 663, and 450 nm), was measured with a spectrophotometer (Arnon, 1949). Total soluble sugars were quantified in oven-dried leaves of *C. aurantium* seedlings using the method of (Malik and Srivastava, 1985). The quantity of proline in *C. aurantium* seedlings was determined using the methods described by Bates et al. (1973). The TPC (total phenolic content) of the samples extract was evaluated using the Folin-Ciocalteu method (Kaur and Kapoor, 2002). The TFC (total flavonoid content) of the crude extract was determined using the aluminum chloride colorimetric method (Chang et al., 2002). TSP (total soluble protein) content of leaves was measured (Bradford, 1976) by comparing it to a standard curve of bovine serum albumin (BSA). These bacteria were isolated and characterized to access their plant growth-promoting traits. However, pH is another important factor to determine the environment or soil pH in which these microbes can naturally grow if applied to the plants in replacement of fertilizers.

### 2.9 Bacterial quantification by real-time PCR

DNA was extracted from infected leaves using the CTAB method (Doyle, 1990) and the samples were then quantified using a Denovox UV spectrophotometer (DS-11). Using 16S rDNA sequences from the database, a primer set for real-time PCR of bacterial endophytes was designed. MEGA 7 was used to perform multiple sequence alignment of the specified bacterial genus using muscle. Primers pair was designed on conserved regions of 16S rDNA i.e., forward primer (GGGGAGCAAACAGGATTAG) and reverse primer (TAAGG TTCTTCGCGTTGCTT). Conventional PCR using Taq polymerase was used to optimize this primer pair. The purified amplified product was then used as a real-time PCR control. The tenfold serial dilution of a standard DNA was accomplished, and 1ul out of each dilution had been used for RT-PCR with qPCR master mix 2X (Thermo fisher scientific, UK). The relative quantification of bacterial cells in testing samples was achieved using an Illumina real-time PCR instrument and the software ECO.

### 2.10 Statistical analysis

All of the acquired data was statistically analyzed using an RCBD (randomized complete block design) with 3 replications. To compare the variations between treatment means, variance analyses were carried out, and means were separated using the least significant difference test (Fisher's LSD) at a 5% level of probability. The complete statistical study was performed with the help of the software package statistics 8.1.

## 3 Results

Bacterial strains that have been identified using morphology and the molecular marker 16S rDNA are shown in Table 1. Strains SM-1, SM-57 showed a 97% sequence identify with *Staphylococcus haemolyticus* and Pseudomonas sp. while SM-42, SM-56, and SM-68 showed sequence identity with *Brevibacillus borstelensis (93%)*, *Bacillus megaterium* (94%), and *Pseudomonas aeruginosa* (95%) respectively. SM-27 and SM-36 give a maximum of 97% sequence identity with *Enterobacter* hormaechei *and Bacillus cereus.* SM-20 and SM-76 give similarity with *Proteus mirabilis* (99%) and *Enterococcus faecalis* (100%) respectively.

### 3.1 Phylogenetic analysis of isolates based on 16S rRNA sequences

**Fig. 1:**
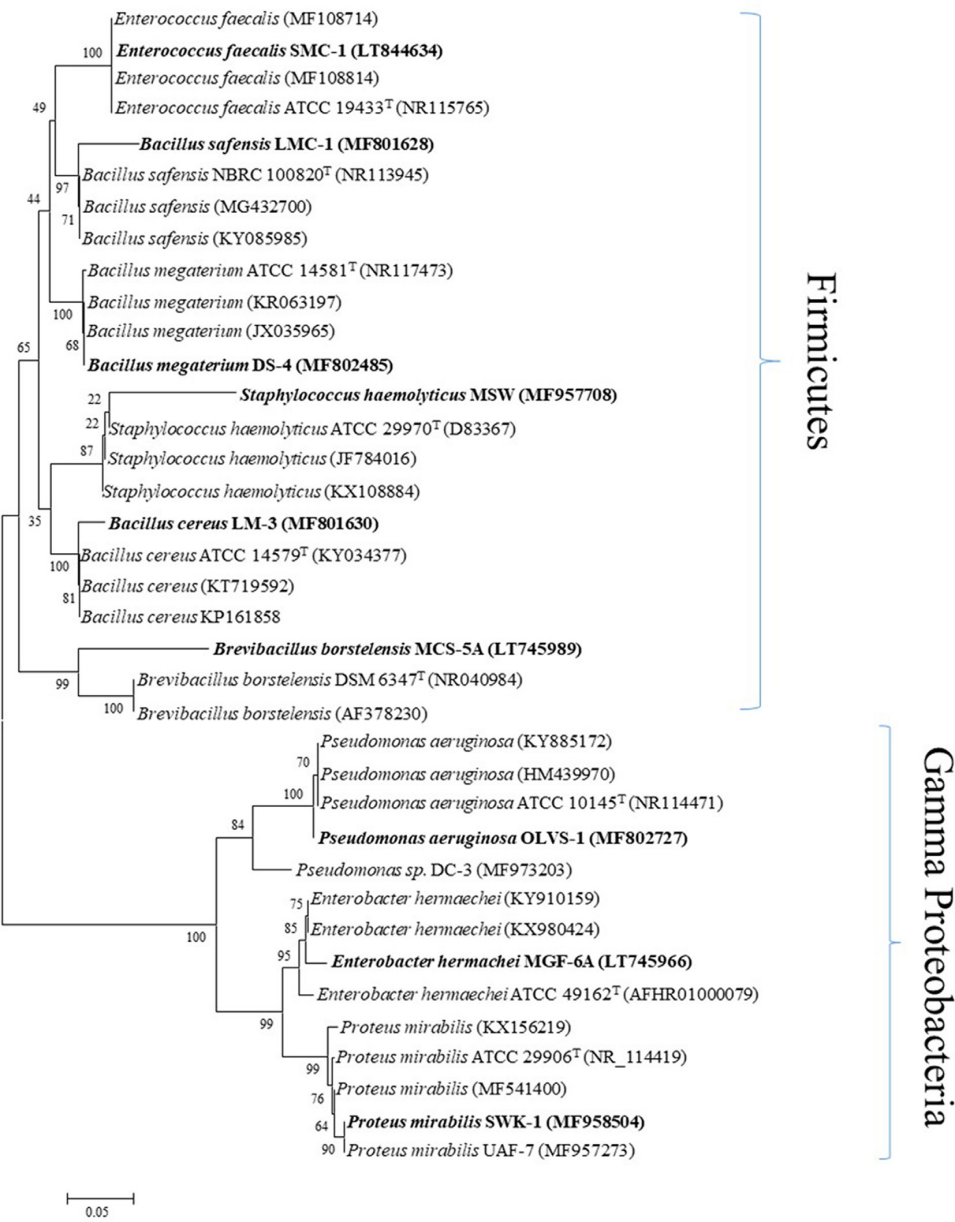
Neighbor-joining phylogenetic tree of bacterial endophytes isolated from leaves of different citrus varieties.

Phylogenetic investigations were performed on all isolates with a nucleotide sequence identity of at least 93–100%. MEGA 6 software and a 1000 bootstrap value was used to create a neighbor-joining dendrogram (Fig. 1). The sequences isolated from citrus are represented by highlighted and bold branch nodes, while others display published sequences from the NCBI database that were utilized to compare results. Phylogenetic analysis grouped isolates into separate clades belonging to the Firmicutes class (e.g., *Staphylococcus haemolyticus*; *Enterococcus faecalis*; *Bacillus safensis*; *Bacillus megaterium*; *Bacillus cereus*; *Brevibacillus borstelensis*). Other Gama Proteobacteria members (*Pseudomonas sp*., *Pseudomonas aeruginosa*, *Enterobacter* hormaechei, and *Proteus mirabilis*) belong to a distinct clade with genetic similarities among strains as compared to previously described strains.

### 3.2 Glasshouse experiment

#### 3.2.1 Effect of inoculation on morphological traits

Inoculation of the citrus (sour orange) rootstock with bacterial strains at the seedling stage (about 1-year-old) considerably improved seedling vigor, according to the results of the pot experiment. In all test methods, all isolates significantly boosted the shoot fresh weight of sour orange seedlings when compared to the control (p < 0.05) (Table 2, Table 3, Table 4). The highest rise in SFW was seen in sour orange seedlings treated with *Pseudomonas* sp. by injecting bacterial cell suspension into the leaves (3.623 g). *Pseudomonas* sp. produces the highest SFW (3.51 g) when inoculated using the spray method. *Bacillus cereus* had the highest SFW when a bacterial suspension was mixed in soil (4.036 g).

**Table 2:** Morphological and physical parameters studied after inoculation by injecting bacterial suspension.

Treatments	SFW (g)	RFW (g)	SDW (g)	RDW (g)	SL (cm)	RL (cm)	RLWC (%)
Un-inoculated Control	1.38ef	0.64h	0.63g	0.43h	33.33cd	14cd	64.64a
*Bacillus safensis*	2.75b	1.18d	0.96e	0.64c	43.33a	16.3c	29.032b
*Pseudomonas* sp.	3.62a	1.36a	1.93b	0.75b	40b	17.13bc	77.407b
*Enterococcus faecalis*	3.15b	1.205c	1.633c	0.76b	31de	19.083a	11.46b
*Bacillus megaterium*	2.043cd	1.195e	1.53c	0.78b	34.66cd	18.25ab	56.53b
*Pseudomonas aeruginosa*	1.19e	0.66h	0.583g	0.32e	36.83c	14.333d	25.37h
*Brevibacillus borstelensis*	1.61e	0.64f	1.013de	0.446d	23.41g	12.41ef	17.39b
*Staphylococcus haemolyticus*	0.89f	0.54i	0.606g	0.45d	28.58f	9.5g	12.655b
*Enterobacter* hormaechei	0.74f	0.54j	0.363h	0.308e	19.16h	16.41c	47.97b
*Bacillus cereus*	2.033d	0.96e	1.127d	3.141c	31.41g	12.166f	86.792b
*Proteus mirabilis*	1.116f	0.556h	0.755f	0.421d	16.75h	12.916d-f	22.365b
Fisher’s LSD	8.418	6.244	0.1243	7.45	2.722	1.639	18.92

The results are the average of three replicates (n = three). Values in the same column accompanied by lower-case letters do not differ significantly (P <0.05) as per Fisher's least significant difference (LSD) method.

**Table 4:** Morphological and physical parameters studied after inoculation by injecting bacterial suspension by saturating the soil with a bacterial suspension (10^8^ CFU).

Treatments	SFW (g)	SDW (g)	RFW (g)	RDW (g)	RLWC (%)	SL (cm)	RL (cm)
Uninoculated control	1.38ef	0.63g	0.64h	0.43h	64.64a	33.33cd	14cd
*Pseudomonas* sp.	2.156ef	1.5003f	0.7e	0.54d	85.49b	37b	17b
*Enterococcus faecalis*	1.92d	1.103g	0.83f	0.46e	14.81b	35.25bc	20.91a
*Bacillus megaterium*	1.45de	0.72g	0.75g	0.454f	66.31b	25.5e	12.416efg
*Enterobacter* hormaechei	0.943f	0.77j	0.631k	0.375f	47.97b	29.41d	14.083cde
*Brevibacillus borstelensis*	1.07g	0.603k	0.36j	0.231g	17.39b	18.25g	8.41h
*Staphylococcus haemolyticus*	0.447g	0.315k	0.35i	0.236i	31.13b	22.13f	19.65a
*Bacillus safensis*	2.84c	1.97e	0.97c	0.65b	68.054b	42.5a	11.5g
*Pseudomonas aeruginosa*	1.12f	0.52i	0.61i	0.39h	35.402b	25.5e	12.58defg
*Bacillus cereus*	4.036c	1.986a	1.516c	3.141b	56.886b	31.41a	13.75fg
*Proteus mirabilis*	2.343b	1.2406d	1.134d	0.672c	22.365b	22.55a	14.35bcd
Fisher’s LSD	1.528	4.04	7.38	1.136	5.94	4.76	6.92

Values in the same column accompanied by lower-case letters do not differ significantly (P <0.05) as per Fisher's least significant difference (LSD) method.

**Table 3:** Morphological and physical parameters studied after inoculation a bacterial suspension (10^8^ CFU) was sprayed on the leaf's surface.

Treatments	SFW (g)	SDW (g)	RFW (g)	RDW (g)	SL(cm)	RL (cm)	RLWC (%)
Control (−ve)	1.38ef	0.63g	0.64h	0.43h	33.33cd	14cd	64.64a
*Bacillus safensis*	2.20cde	1.84c	0.83c	0.65b	44a	12.5de	52.58b
*Pseudomonas* sp.	3.51bc	1.43e	1.03a	0.56c	33.33cd	15.5cd	29.98b
*Enterococcus faecalis*	1.95a	1.356d	0.76f	0.62c	38.75b	16.41bc	15.54b
*Bacillus megaterium*	1.51de	1.128cd	0.65e	0.56d	37.583b	10.35e	34.057b
*Pseudomonas aeruginosa*	1.803ef	1.45e	1.41g	0.614e	36.1bc	14.086cd	72.22b
*Brevibacillus borstelensis*	0.87ef	0.616f	0.683j	0.492hi	14.33g	14.6cd	17.39b
*Staphylococcus haemolyticus*	1.05bcd	0.543e	0.95i	0.576i	24.5e	22a	81.78b
*Enterobacter* hormaechei	2.105b	1.0028cd	1.125d	0.627f	33.75cd	18.916b	80.780b
*Bacillus cereus*	2.226a	1.159b	1.443c	3.141e	31.41d	15.766c	57.522b
*Proteus mirabilis*	1.56f	0.9006h	0.523g	0.315g	21.75f	12.41de	39.89b
Fisher’s LSD	0.185	3.32	5.0432	7.629	2.615	2.775	18.927

Values in the same column accompanied by lower-case letters do not differ significantly (P <0.05) as per Fisher's least significant difference (LSD) method.

In all test methods, several isolates significantly boosted the root fresh weight of sour orange seedlings when compared to the control (p < 0.05). *Bacillus megaterium* (1.193 g) demonstrated the highest RFW when inoculated by injection, while *Enterococcus faecalis* showed the highest RFW when inoculated by spray (1.413 g). *Bacillus cereus*, on the other hand, produced greater RFW (1.516 g) in the soil mix method. *Pseudomonas sp*. gave the highest rise in SDW (1.921 g) in the injection method, while *Enterococcus faecalis* gave the lowest SDW in the spray method (1.456 g). *Bacillus cereus*, on the other hand, showed reduced SDW in the soil mix treatment (1.986 g). The RDW of sour orange seedlings exhibited statistically significant findings, with *Bacillus cereus* showing the highest RDW (3.141 g) in the injection method and *Bacillus safensis* showing the lowest RDW (0.653 g) in the spray method, as compared to a positive control (0.872 g). *Bacillus cereus* had a lower RDW when using the soil mix method (0.652 g). When sour orange seedlings were compared to the control, the shoot/root length exhibited statistically significant results. *Bacillus safensis* demonstrated SL (43.33 cm) in the injection method and SL (43.33 cm) in the spray method.

When compared to the positive control, *Bacillus safensis* demonstrated less SL (42.5 cm) in the soil mix method (44.16 cm). In the injection method, *Enterococcus faecalis* exhibits the highest RL (19.083 cm). In the spray method, *Staphylococcus haemolyticus* exhibited a higher RL of (22 cm), but in the soil mix methodology, *Enterococcus faecalis* showed a higher RL of 16.41 cm, compared to the positive control of (14.5 cm). In comparison to control, the relative leaf water content (%) of sour orange seedlings demonstrated statistically non-significant results. *Bacillus cereus* had the highest RLWC (86.79 %) compared to a positive control (23.48%) in the injection method, while surface *Staphylococcus haemolyticus* had the highest RLWC in the spraying method (81.783 %). In the soil mix method, *Pseudomonas sp*. had the highest RLWC (85.49%) when compared to a positive control (23.48%).

#### 3.2.2 Physiological parameters of sour orange seedlings

In vitro tests for total soluble sugars in sour orange seedlings (mg/g fresh weight) were performed on the bacterial isolates (Table 5, Table 6, Table 7). In all of the test techniques, the TSS exhibited statistically significant outcomes when compared to control (p < 0.05), whereas the remaining isolates had no significant effect on seedling TSS when compared to control. In the injection method, *Proteus mirabilis* had a higher total soluble sugar content (23.29 mg/g). *Bacillus cereus* provided a higher TSS (22.37 mg/g) in the spray method, whereas *Proteus mirabilis* gave more TSS (22.023 mg/g) in the soil mix method, compared to a positive control (22.96 mg/g). In the spray and injection methods, the chlorophyll (a) of sour orange seedlings showed statistically significant results when compared to control (p < 0.05), but not in the soil mix. *Staphylococcus haemolyticus* produced more chlorophyll (a) (0.2201 mg/g) compared to a positive control (0.1854 mg/g) in the injection method, while *Bacillus cereus* produced more chlorophyll (a) (0.196 mg/g) in the spray method. The chlorophyll (b) of sour orange seedlings demonstrated statistically significant results when compared to control (p < 0.05) in the soil mix method, but non-significant results when compared to control in the spray & injection method. *Bacillus safensis* had higher chlorophyll (b) (3.976 mg/g) in the soil mix method, but *Enterobacter* hormaechei had a minimum of (0.837 mg/g).

**Table 5:** Physiological parameters studied after inoculation a bacterial suspension (108 CFU) was injected into the backside of the leaf.

Treatments	Proline	Protein	Phenolic	Flavonoids	Chlorophyll *a*	Chlorophyll *b*	Carotenoids	TSS(mg/g)
Uninoculated control	11.64ab	2.65d	1.35a	15.42a–c	0.15a	3.45a	9.68bc	22.96a
*Bacillus safensis*	13.50ab	0.76i	1.59a	16.28ab	0.16a	3.34ab	8.54b–d	10.85c
*Pseudomonas* sp.	12.61ab	2.34e	1.487a	15.47ab	0.132a	2.92a–c	8.48b–d	12.56c
*Enterococcus faecalis*	15.39a	2.92c	1.11a	8.68c	0.106a	1.607bc	8.605b–d	15c
*Bacillus megaterium*	10.63ab	2.44de	1.34a	8.12c	0.16a	2.65a–c	11.35b	15.02bc
*Pseudomonas aeruginosa*	7.004b	1.86fg	0.97a	8.42c	0.15b	1.49c	4.19e	20.13a
*Brevibacillus borstelensis*	8.29ab	1.69g	1.30a	16.81ab	0.13a	2.04a–c	6.51de	21.75a
*Staphylococcus haemolyticus*	6.58b	1.08h	1.32a	10.41bc	0.22a	2.63a–c	9.42b–d	20.66a
*Enterobacter* hormaechei	8.10ab	2.05f	1.49a	15.60ab	0.15a	3.12a–c	8.50b–d	18.21ab
*Bacillus cereus*	8.46ab	4.82a	1.71a	12.15bc	0.13a	2.18a–c	8.68cd	18.62ab
*Proteus mirabilis*	6.76b	4.76a	0.86a	18.43a	0.096ab	4.28a	4.42e	23.29a
Fisher’s LSD	4.62	6.28	6.26	2.67	0.262	0.076	1.574	0.782

Values in the same column accompanied by lower-case letters do not differ significantly (P <0.05) as per Fisher's least significant difference (LSD) method.

**Table 6:** Physiological parameters studied after inoculation a bacterial suspension (10^8^ CFU) sprayed on the leaf's surface.

Treatments	Proline	Protein	Phenolic	Flavonoids	Chlorophyll *a*	Chlorophyll *b*	Carotenoids	TSS (mg/g)
Uninoculated control	11.64ab	2.65d	1.35a	15.42a–c	0.15a	3.45a	9.68bc	22.96a
*Bacillus safensis*	13.85ab	2.30ab	1.43ab	19.29c	0.11b–e	3.91a	6.51cde	15.27b
*Pseudomonas* sp.	10.47ab	2.02ab	1.52a	19.10a	0.12a-d	2.21d	6.48cde	15.37b
*Enterococcus faecalis*	10.30ab	2.87ab	1.16ab	8.20bc	0.04de	2.01cd	5.35de	14.6b
*Bacillus megaterium*	10.02ab	0.98ab	1.26ab	11.06a–c	0.03e	1.65d	7.34cd	22.02a
*Pseudomonas aeruginosa*	5.39b	2.22ab	1.62a	11.11a–c	0.07c–e	1.71d	11.21b	21.35a
*Brevibacillus borstelensis*	12.79b	2.9ab	1.60a	16.61a	0.14a–c	3.53a-c	8.31b-d	19.92a
*Staphylococcus haemolyticus*	6.62ab	3.4ab	0.82b	18.89a	0.10bc–e	2.03cd	9.62bc	15.73b
*Enterobacter* hormaechei	7.38ab	1.26b	1.10ab	16.22a	0.14a-c	3.24a–d	6.60c-e	18.69ab
*Bacillus cereus*	5.16b	4.42a	1.60a	17.22a	0.19a	2.60cd	3.83e	22.37a
*Proteus mirabilis*	5.94b	4.12a	1.54a	16.44a	0.17ab	3.79a–d	9.20bc	21.03a
Fisher’s LSD	7.47	2.37	0.53	7.11	7.17	1.47	2.88	3.93

Values in the same column accompanied by lower-case letters do not differ significantly (P <0.05) as per Fisher's least significant difference (LSD) method.

**Table 7:** Physiological parameters studied after inoculation by mixing bacterial suspension (10^8^ CFU) on the soil.

Treatments	Protein	Proline	Chlorophyll *a*	Chlorophyll *b*	Carotenoids	TSS(mg/g)	Phenolic	Flavonoids
Uninoculated control	2.65d	11.64ab	0.15a	3.45a	9.68bc	22.96a	1.35a	15.42a–c
*Bacillus safensis*	2.26f	11.49a–c	0.13ab	3.97a	9.67bc	21.34a	1.72a	12.86b–d
*Pseudomonas* sp.	1.55h	12.06ab	0.14ab	3.42abc	9.54bc	13.91a	1.59ab	14.80a–d
*Enterococcus faecalis*	2.29f	7.807bc	0.11ab	2.62abcde	6.64d–f	18.52a	1.13a–d	7.77e
*Bacillus megaterium*	2.45e	8.66bc	0.14ab	1.76ef	5.67ef	21.15a	1.1003a–d	7.15e
*Pseudomonas aeruginosa*	1.58h	7.13bc	0.19ab	2.59a–e	8.03c–e	20.53a	1.38a–c	13.16b–d
*Brevibacillus borstelensis*	1.12j	5.75c	0.13ab	2.19cd–f	11.04b	21.24a	0.69b–d	10.81c-e
*Staphylococcus haemolyticus*	2.07g	6.89bc	0.084ab	3.31a–d	6.75d–f	8.85a	0.26d	10.41de
*Enterobacter* hormaechei	1.23i	6.05bc	0.044b	0.83f	2.35g	19.19a	0.63cd	13.46b–d
*Bacillus cereus*	4.27b	5.54c	0.204a	2.46d–f	4.21fg	21.71a	1.63ab	11.96a–c
*Proteus mirabilis*	5.15a	6.86bc	0.15a	1.81a–e	8.50cd	22.02a	1.30a–c	21.41ab
Fisher’s LSD	7.48	5.30	9.0089	1.24	2.36	3.82	0.83	4.33

Values in the same column accompanied by lower-case letters do not differ significantly (P <0.05) as per Fisher's least significant difference (LSD) method.

The carotenoids in sour orange seedlings exhibited statistically significant findings for all isolates when compared to the control (p < 0.05 in all test methods). *Bacillus cereus* produced more carotenoids (4.21 mg/g) in the soil mix method, while *Bacillus megaterium* produced more carotenoids (3.832 mg/g) in the spray method, and *Bacillus cereus* produced more carotenoids (11.355 mg/g) in the injection method. When compared to control, the protein content of sour orange seedlings showed statistically significant results for injection and soil mix methods, but non-significant results for spray method of all isolates at (p < 0.05). *Bacillus cereus* produced more proteins (4.829 mg/g) in the injection method, while *Proteus mirabilis* produced more proteins (5.155 mg/g) in the soil mix method.

The phenolic content (mg GAE/g) of sour orange seedlings was determined in vitro using bacterial isolates. The phenolic contents of sour orange seedlings showed statistically significant results for the soil mix and spray methods, but non-significant results for the injection method, when compared to the control. In the spray method*, P. aeruginosa* had more phenolics (1.626 mg GAE/g), whereas, in the soil mix method, *Proteus mirabilis* had more phenolics *(*1.7226 mg GAE/g).

The proline content of sour orange seedlings was statistically significant in contrast to control for soil mix and injection methods, but non-significant for spray methods. *Staphylococcus haemolyticus* had a higher proline content of (6.585 µg/g) fresh weight in the injection method, while *Pseudomonas* sp. had a higher proline content of (12.064 µg/g) in the soil mix method. In all test methods, the flavonoid content of sour orange seedlings revealed statistically significant results when compared to control (p < 0.05). *Proteus mirabilis* had a higher flavonoid content of (18.431 mg (QE)/g) in the injection method, while *B. safensis* had a higher flavonoid content of (19.298 mg (QE)/g) in the spray approach. *Proteus mirabilis*, on the other hand, showed higher proteins in the soil mix method (21.417 mg (QE)/g).

#### 3.2.3 Mix infection of test bacterial strains into sour orange seedlings

**Fig. 2:**
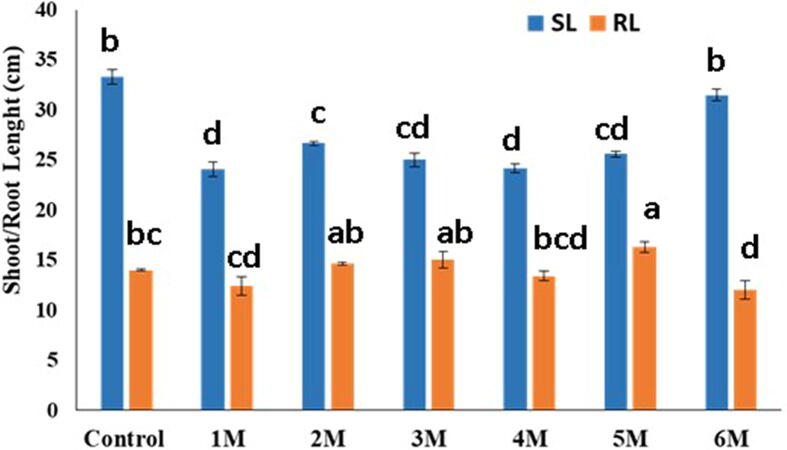
Morphological parameters (Shoot/Root length) studied after inoculation by mixing bacterial suspension (10^8^ CFU) on the soil.

**Fig. 3:**
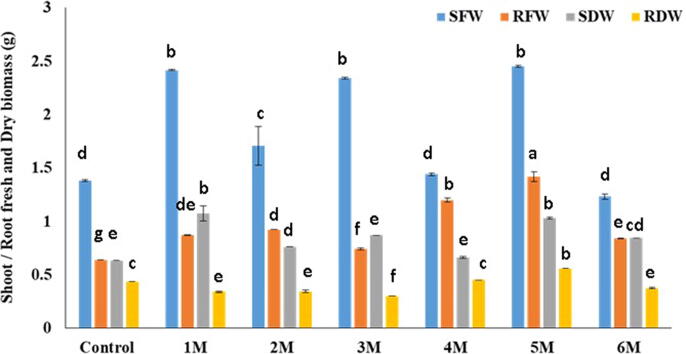
Morphological parameters (Shoot/Root fresh and dry biomass) studied after inoculation by mixing bacterial suspension (10^8^ CFU) on the soil.

**Fig. 4:**
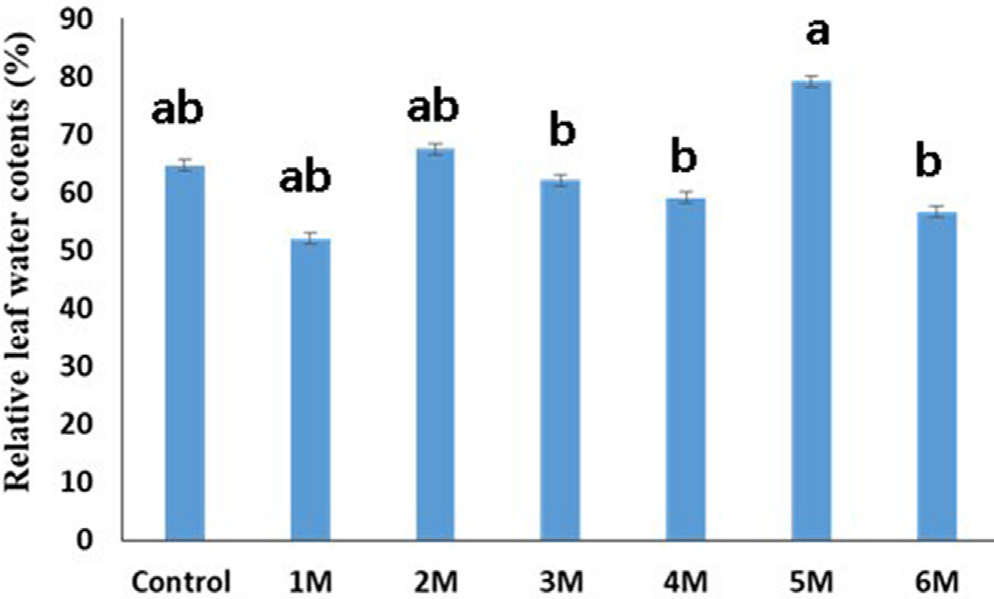
Morphological parameters (Relative leaf water contents) studied after inoculation by mixing bacterial suspension (10^8^ CFU) on the soil.

**Fig. 5:**
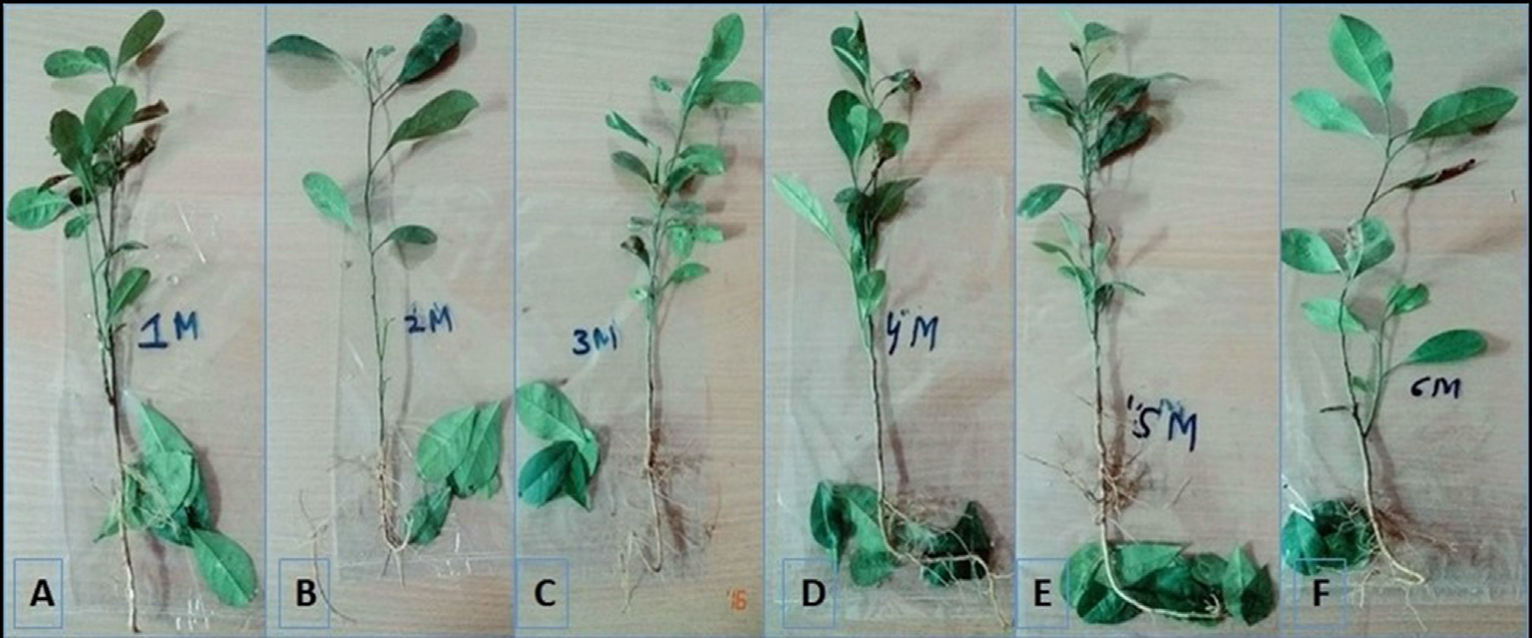
Illustration of sour orange seedlings infected with bacterial endophytes isolated from citrus. (A) SM-34 + SM-57 + SM-56 + SM-68, (B) SM-34 + SM-57 + SM-56 + SM-42, (C) SM-34 + SM-57 + SM-56 + SM-20, (D) SM-34 + SM-57 + SM-56, (E) SM-68 + SM-42 + SM-36 + SM-20, (F) SM-42 + SM-68 + SM-56 + SM-20.

To investigate the interaction of several bacteria in citrus plants, the isolates that showed better results in terms of beneficial influence on seedlings in the first experiment were mixed and applied to sour orang seedlings as follows: 1 M (*Pseudomonas* sp.*, B. megaterium, B. safensis, P. aeruginosa*); 2 M (*Pseudomonas* sp.*, B. safensis, P. aeruginosa, B. megaterium*); 3 M (*B. megaterium, Pseudomonas* sp.*, Proteus mirabilis., B. safensis*); 4 M (*Pseudomonas* sp.*, B. safensis, B. megaterium, B. cereus*); 5 M (*Pseudomonas* sp.*, B. safensis , B. megaterium*); 6 M (*B. cereus, Brevibacillus borstelensis, Proteus mirabilis, P. aeruginosa*). The bacterial isolates were tested in vitro to assess how a mixed bacterial inoculum would affect the sour orange seedling. The SL/RL length of sour orange seedlings displayed statistically significant results when compared to the control for all strains (p < 0.05). The syringe method was used to apply all of the treatments for mixed infection to the sour orange seedling leaf (Fig. 2, Fig. 3, Fig. 4, Fig. 5).

As per findings, the maximum SL in control was (44.16 cm), while the minimum SL in 1 M was (24.08 cm). Similarly, (14.5 cm) were found in control, (16.33 cm) in 5 M, which was higher than control, and a minimum of (12 cm) in 6 M, correspondingly. When compared to the control, all of the isolates had statistically significant results for fresh and dry biomass (g) of sour orange seedlings (p < 0.05). The maximum value of SFW was (3.356 g) in control and (2.45 g) in 5 M. The highest RFW in 5 M was (1.42 g) against (1.1136 g) in the control. Control had a maximum SDW of (2.176 g), while 1 M had a maximum SDW of (1.073 g). The maximum RDW was (0.872 g) in the control and (0.5596 g) in the 5 M. The maximum RLWC reported in 5 M was (79.15 %), compared to (23.48 %) in the control.

#### 3.2.4 Physiological traits of seedlings inoculated with a mixed infection

In 5 M *Bacillus safensis*, *Pseudomonas sp*., and *Bacillus elaterium*, the maximum value of proteins was (5.03 mg/g) fresh weight, compared to (3.21 mg/g) fresh weight in control (Table 8). The maximum phenolic concentration was found to be (1.627 mg GAE/g) in 5 M, compared to (1.723 mg GAE/g) in the control. In 5 M, the maximum flavonoids were (18.425 mg (QE)/g), compared to (18.846 mg (QE)/g) in the control. In 2 M, the maximum TSS was (0.1986 mg/g), compared to (2.218 mg/g) in healthy controls. In comparison to control (0.1854 mg/g), maximum chlorophyll *a* contents were (0.1983 mg/g) in 6 M and (0.1937 mg/g) in 4 M. In 6 M, the maximum chlorophyll *b* content was (4.474 mg/g), and in 4 M, it was (4.366 mg/g), compared to (3.766 mg/g) in the control. In comparison to control, the maximum carotenoid content was (8.829 mg/g) in 6 M and (8.591 mg/g) in 1 M. In 4 M, (14.493 mg/g) maximum proline level was (7.62 mg/g), compared to (8.475 mg/g) in control.

**Table 8:** Physiological parameters studied after inoculation by injecting bacterial suspension mix infection of different bacterial inoculums*.*

Treatments	Proline	chlorophyll a	Chlorophyll b	Carotenoids	TSS	Phenolic	Flavonoids
Control	11.64a	0.15b	3.45e	9.68b	2.29a	1.35a	15.42b
1M	7.51b	0.091e	4.107c	8.59bc	0.085cd	1.39a	12.07c
2M	6.39b	0.074f	4.23bc	6.61cd	0.19b	1.44a	10.85c
3M	6.84b	0.073f	4.25bc	4.73d	0.084cd	1.32a	11.84c
4M	7.62b	0.103cd	4.36ab	6.33cd	0.024d	1.42a	9.76c
5M	7.018b	0.098de	4.18c	7.00b-d	0.13bc	1.62a	18.42ab
6M	6.91b	0.108c	4.47a	8.82bc	0.12bc	1.48a	18.35ab
Fisher’s LSD	2.3815	7.03129	0.1498	2.7072	8.3577	0.3621	3.0616

Values in the same column accompanied by lower-case letters do not differ significantly (P <0.05) as per Fisher's least significant difference (LSD) method.

### 3.3 Bacterial quantification using real-time PCR

The number of bacterial cells growing on sour orange leaves was determined using a 16S rDNA-gene copy number of bacterial endophytes in order to determine the rate of competition between both injected endophytes and the indigenous microbial populations. The results show a slight increase in the colonization of bacterial endophytes on the leaves of the infected treatment as compared to the healthy plants (Table 9). The application of bacterial cells may have resulted in an increase in bacterial CFU in treated plants. The rise in the number of bacterial cells in inoculation treatments suggests that these imported bacterial strains were successfully colonized and also its impact on the structure of the indigenous bacterial population. In this study, the Syber green/Rox qPCR master mix was used to compare the number of bacterial cells in treated vs untreated plants (Table 9). Real-time PCR calibration curves were linear, with correlation coefficients ranging between 0.99 and 1.00. These findings reveal that using original undiluted DNA samples, it is possible to count the copy numbers of bacteria's target 16S rDNA genes in plant tissues. Without triggering the plant defense mechanism, the inoculants were able to compete successfully with the normal bacterial population prevalent in plant tissues. This process could be one of the strategies for boosting plant development.

**Table 9:** Relative quantification of injected bacterial strains 10^8^ cell/plant in sour orange after two weeks.

Treatments used for this study	Inoculation methods	Relative quantity of bacterial endophytes
Positive Control	Injected with ddH_2_O	2.49 × 10^14^ ± 0.08

*Proteus mirabilis*	Soil mixing	7.74 × 10^6^ ± 0.65
	Spray method	3.19 × 10^8^ ± 0.06
	Injection method	0*

*Bacillus cereus*	Soil mixing	0.25 × 10^4^ ± 0.01
	Spray method	0.38 × 10^5^ ± 0.05
	Injection method	0*

*Bacillus safensis*	Soil mixing	0*
	Spray method	9.76 × 10^6^ ± 0.07
	Injection method	0.35 × 10^6^ ± 0.06

*Bacillus megaterium*	Soil mixing	0.09 × 10^5^ ± 0.22
	Spray method	0.37 × 10^6^ ± 0.11
	Injection method	75.69 × 10^6^ ± 1.26

*Staphylococcus haemolyticus*	Soil mixing	0.15 × 10^3^ ± 0.07
	Spray method	0.01 × 10^1^ ± 0.02
	Injection method	0*

*Enterobacter* hormaechei	Soil mixing	0.05 × 10^5^ ± 0.01
	Spray method	0.02 × 10^2^ ± 0.03
	Injection method	1.36 × 10^14^ ± 0.02

*Enterococcus faecalis*	Soil mixing	4.13 × 10^8^ ± 0.08
	Spray method	2.56 × 10^6^ ± 0.06
	Injection method	15.39 × 10^9^ ± 1.21

*Pseudomonas aeruginosa*	Soil mixing	0.62 × 10^4^ ± 0.03
	Spray method	0*
	Injection method	10158.94 × 10^2^ ± 1.56

*Pseudomonas* sp.	Injection method	0*
	Spray method	1.53 × 10^5^ ± 0.02
	Soil mixing	2.47 × 10^9^ ± 0.05

*Brevibacillus borstelensis*	Soil mixing	0.15 × 10^3^ ± 0.06
	Spray method	0.21 × 10^3^ ± 0.03
	Injection method	0*

Note: Expression as x-fold rise in injected endophytic bacteria in treated sample in comparison to the healthy (mean of 3 replications, SD.

* Below the detection limit of 54 copies per microlitre (µl).

## 4 Discussion

Plants are frequently subjected to different types of biotic and abiotic stressful events that exist under complex environmental situations (Zhang et al., 2015). Bacterial infections are one of the most common biotic stresses that impact plant growth, and as a result, agricultural yield losses occur (Denancé et al., 2013). Despite the fact that major scientific efforts have been focused on plant-bacterial interactions. Several studies have been conducted to explore the effects of bacterial inoculum on numerous plant hosts, but little is known about inoculum methods and their influence on host physiological functions. A comparison of several inoculating strategies on the physiology of sour orange seedlings was investigated in the present study. Wu et al., (2005) demonstrated the plant growth-promoting effects of PGPR strains in different crops. Bacterial inoculants can boost seedling emergence, increase plant development and germination, adapt to stress factors, and protect plants from diseases (Lugtenberg et al., 2002). Bacterial endophytes (Pseudomonas, Azotobacter, Azospirillum, Bacillus, and Azomonas) are now widely used as bio-inoculants to promote plant growth and development under a range of different stresses, including heavy metals (Ma et al., 2011), herbicides, insecticides, fungicides (Ahemad and Khan, 2012).

Sugarcane plants infected with endophytes in the field dramatically boosted plant height and shoot length, according to the literature. *Bacillus* spp. and *Pseudomonas spp*. bacterial strains have been found to increase plant development in grape wine, tomato, maize, rice, and sugar beet through a variety of ways (Mehnaz, 2011, Wang et al., 2009). Pseudomonas and Azospirillum have been shown to have agricultural potential and could be used as natural fertilizers (Çakmakçi et al., 2006). Inoculation of plants with Azospirillum caused significant modifications in numerous growth parameters, including plant biomass, nutrient uptake, tissue N content, plant height, leaf size, and root length of cereals (Bashan et al., 2004).

Rhizobacteria have been shown to improve seed germination parameters in a variety of plants, including pearl millet (Niranjan-Raj et al., 2004), maize (Egamberdiyeva, 2007), sugar beet (Çakmakçi et al., 2006), wheat, and sunflower (Salantur et al., 2006). Vikram et al. (2007) found that inoculating *Piper nigra* plants with PGPR increased root length compared to a control, which is similar to our findings. Other researchers have documented improvements in various crop plant growth metrics as a result of PGPR inoculation (Gravel et al., 2007). According to Akbari et al. (2007), wheat seedling roots responded positively to bacterium injection by increasing root length, dry weight, and lateral root hairs.

Kıdoglu et al. (2008) reported that inoculation with PGPR increased the growth of cucumber, tomato, and pepper seedlings. It was reported that PGPR applications increased shoot weight, shoot length, and stem diameter of muskmelon and watermelon seedlings (Kokalis- Burella et al., 2003). García et al. (2003) investigated the impact of PGPR on tomato and pepper seedling growth in various combinations. Leaf relative water contents (LRWC) are a significant replacement for measuring plant water status and hence serve as an indicator of metabolic activity inside cells (Seghatoleslami et al., 2008). Atteya, 2003, Siddique et al., 2000 found similar results with drastically altered internal water status of maize under drought due to a decrease of water potential and LRWC; therefore this affected the photosynthetic rate and reduced the crop yield. The chlorophyll content of citrus seedlings was reduced after inoculation with a single bacterial strain, but it did not affect seedlings treated with a mixture of more than two bacterial strains, indicating that more than two strains could work synergistically during the plant's growth and progression. This is comparable to the findings of Yu et al. (2014), who discovered that co-inoculation of *Pseudomonas aurantiaca*, *Pseudomonas fluorescens*, and *Bacillus cereus* on walnut (*Juglans siggillata* L.) seedlings boosted net photosynthetic rate. In comparison to individual inoculation, co-inoculation of the three strains enhanced the chlorophyll content of the seedlings. This could be due to increased photosynthetic activity as a result of increased N incorporation, which contributes to the creation of chlorophyll content (Liu et al., 2013).

Under biotic and abiotic stress conditions, total soluble sugars (TSS) play a complex role within cells. They could function as metabolic regulatory signal molecules (Gibson, 2005). According to our results, the level of TSS was affected by the single inoculum while it was reduced a bit in mixed bacterial inoculums. The rise in carbohydrates may benefit plant metabolism under stressed conditions, maintenance of energy or carbon supply, and plant homeostasis (Vargas et al., 2014). Phenolic compounds serve as signaling molecules in the establishment of symbioses and also act as plant defense agents. Flavonoids are a group of polyphenolic compounds that have gained a lot of interest due to their role as signaling molecules in plant–microbe interactions. By chelating trace components involved in the free-radical synthesis, flavonoids scavenge reactive species, reduce reactive oxygen synthesis, and up-regulate as well as sustain antioxidant defenses (Agati, et al., 2012). Flavonoids have also been shown to prevent plant pathogen spore germination (Harborne and Williams, 2000). Polyphenol toxicity for bacteria may be caused by the inhibition of hydrolytic enzymes (proteases) or other associations that inhibit the growth of microbial adhesions, cell envelope transport proteins, and non-specific interactions with carbohydrates (Pyla et al., 2010). The total phenolic concentration could be used as a basis for quick screening of antioxidant activity since their hydroxyl group aid in free radical scavenging. Flavonoids, which include flavones, flavonols, and condensed tannins, are secondary metabolites found in plants whose antioxidant activity is dependent on the supply of free OH groups, mainly 3-OH (Bose et al., 2018).

The significance of proline in the efficient survival of plants under stress situations is complex and diversified. Proline may also contribute to the preservation of protein structures within the cell. Proline also plays a significant role in the activities of various enzymes, the regulation of cell pH, and antioxidant properties by scavenging (ROS) (Verbruggen and Hermans, 2008). Plants accumulate massive proline quantities under stressful situations. Plants that have been inoculated with PGPRs accumulate more osmolytes. When plants were injected with *Pseudomonas mendocina*, its abundance increased significantly (Kohler et al., 2008). The quantity of protein declines as the stress continues, due to a drastic reduction in photosynthesis or a lack of raw materials for protein synthesis, resulting in a significant decline or even complete termination of the process (Mohammad Khani and Heidari, 2008). Protein degradation is accelerated due to increased activity of protease or other catabolic enzymes, which activate during stress. These changes might be due to protein crumbling caused by toxic effects of reactive oxygen species, leading to lower protein content and characteristic symptom of oxidative stress have been observed in stressed plants (Seel et al., 1992, Moran et al., 1994). A similar trend was observed in the current study, but the use of the bacterial inoculum attenuated the impacts by creating a significant reduction in protein quantities under stressed conditions.

## 5 Conclusion

Phyllosphere bacterial endophytes also have some potential as plant growth-promoting activity as well as they can be used as biological control agents. There is a need to check these bacterial inoculants in sour orange seedlings under field conditions. There is much literature about abiotic stress and its effects on plant physiological functioning. But, insufficient literature is available on the influence of bacterial inoculants on physiological aspects of plants. Our findings suggest that bacterial inoculants influence sour orange seedling physiology and aid in the enhancement of physicochemical characteristics. These physical responses are unrelated to biomass production in plants, even though mixed infection with bacterial endophytes improves seedling vegetative growth. The author suggests that after thorough experimentation it was found that the injection or soil mix method is good for inoculation of endophytes as PGPRs. Our strains *B. megaterium, B. cereus, B. safensis, Proteus mirabilis,* and *P. aeruginosa* produce good results to improve plant growth in both isolated and combined inoculation. Suggested that these inoculants could be used as biocontrol agents and plant growth promoters in replacement of harmful chemical fertilizers.

## Declaration of Competing Interest

The authors declare that they have no known competing financial interests or personal relationships that could have appeared to influence the work reported in this paper.
